# *FOXD3* may be a new cellular target biomarker as a hypermethylation gene in human ovarian cancer

**DOI:** 10.1186/s12935-019-0755-8

**Published:** 2019-02-28

**Authors:** Gui-fang Luo, Chang-ye Chen, Juan Wang, Hai-yan Yue, Yong Tian, Ping Yang, Yu-kun Li, Yan Li

**Affiliations:** 1 Department of Gynecology, The First Affiliated Hospital of University of South China, Hengyang, 421001 People’s Republic of China; 2 Clinical Anatomy & Reproductive Medicine Application Institute, Department of Histology and Embryology, University of South China, Hengyang, 421001 Hunan People’s Republic of China; 3 Key Laboratory of Tumor Cellular and Molecular Pathology, College of Hunan Province, Cancer Research Institute, University of South China, No. 28 West Changsheng Road, Hengyang, 421001 Hunan People’s Republic of China; 4 Department of Obstetrics and Gynecology, Central Hospital of Enshi Tujia and Miao Autonomous Prefecture, Enshi Clinical College of Wuhan University, Enshi, 445000 Hubei People’s Republic of China; 5 Institute of Reproductive and Stem Cell Engineering, School of Basic Medical Science, Central South University, No. 932 South Lushan Road, Yuelu District, Changsha, 410013 Hunan People’s Republic of China; 6 Reproductive and Genetic Hospital of Citic-Xiangya, No. 84 Xiangya Road, Changsha, 410078 Hunan People’s Republic of China

**Keywords:** Ovarian carcinoma, DNA methylation, 5-Aza-dC, *FOXD3*

## Abstract

**Background:**

*FOXD3* is aberrantly regulated in several tumors, but its underlying mechanisms in ovarian cancer (OC) remains largely unknown. The present study aimed to explore the role and associated mechanisms of *FOXD3* in OC.

**Methods:**

Microarray data from GEO was used to analyze differential CpG sites and differentially methylated regions (DMR) in tumor tissues and Illumina 450 genome-wide methylation data was employed. The *FOXD3* expression level was determined through qRT-PCR and western blot analysis. Wound healing test, colony formation and flow cytometry assay were utilized to analyze cell migration, proliferation abilities, cell cycle and cell apoptosis, respectively. Finally, the effect of *FOXD3* on tumor growth was investigated through in vivo xenograft experiments.

**Results:**

GEO data analysis showed that *FOXD3* was hypermethylated in OC tissues. Also, qRT-PCR revealed that *FOXD3* was low expressed and methylation-specific PCR (MSP) confirmed that the methylation level of *FOXD3* was hypermethylated. Combined treatment of 5-aza-2′-deoxycytidine (5-Aza-dC) could synergistically restored *FOXD3* expression. Finally, in vitro and in vivo experiments showed that demethylated *FOXD3* decreased cell proliferation and migration abilities, and increased the cell apoptosis. In vivo experiment detected that demethylated *FOXD3* restrained tumor growth.

**Conclusions:**

*FOXD3* could act as a tumor suppressor to inhibit cell proliferation, migration and promote cell apoptosis in OC cells.

**Electronic supplementary material:**

The online version of this article (10.1186/s12935-019-0755-8) contains supplementary material, which is available to authorized users.

## Background

Ovarian cancer (OC) is the sixth commonest diagnosed malignancy among women around world, the second most frequent gynecologic cancer, and also the most lethal tumor of female reproductive system [1]. There are three major groups in ovarian cancers: epithelial, germ cell, and specialized stromal cell tumors in which epithelial ovarian cancers (EOCs) are the vast amount of ovarian cancers [2]. Despite the fact that surgical techniques and adjunct therapies have been improved for years, little change has occurred in the survival rate of ovarian cancer since platinum-based therapy was spread over past 30 years [3]. Manifesting chemical resistance, late detection and deficiency of targeted therapies for advanced ovarian cancers are considered to be the primary factor lead to poor prognosis [3]. For these reasons, ovarian cancer has become one of the greatest clinical challenges [4]. In order to develop new drug strategies or diagnostic biomarkers, a better appreciation of the molecular mechanism of ovarian cancer is needed [5].

Since the association between aberrant DNA methylation patterns and malignancy was first found, its role in cancer development has been increasingly investigated [6]. DNA methylation consists of the epigenetic mechanism connected to gene expression. DNA methylation is found on the cytosine residues of CG (CpG) dinucleotides [4]. DNA methyltransferases (DNMTs) could catalyze the adjunction of a methyl group to the cytosine ring to form methyl cytosine in which *S*-adenosylmethionine is recruited as a methyl donor [4]. Both the hypermethylation inducing the silence of tumor suppressor genes and hypomethylation associated with genomic instability have been involved in cancer initiation and tumor progression [7]. For example, aberrant methylation of CpG islands is a common epigenetic event occurred in EOC, which has the most variation type [8]. Therefore, investigation on variation of tumor DNA methylation contributes to better understand the mechanism of cancer.

As a transcriptional factor, *FOXD3* is a member of the forkhead gene family and plays significant roles during development, cell maintenance and regulation of lineage specification [9, 10]. What’s more, FOXD3 plays a significant role in tumor initiation and growth through other transcription factors like TWIST1 [11]. Many studies have revealed the association between *FOXD3* and tumorigenesis. FOXD3 could inhibits non-small cell lung cancer growth [12]. In addition, low expression of *FOXD3* contributes to poor prognosis in high-grade glioma patients [13]. However, the function of *FOXD3* in ovarian cancer is still not explicit, which urges us to clarify its mechanism.

In this study, *FOXD3* was demonstrated that the degree of methylation and expression in various ovarian cancer cells were changed compared to normal ovarian cells. Our results suggested that *FOXD3* could affect tumor growth and aggressiveness in OC.

## Methods

### Bioinformatic analysis

Bioinformatic analysis was based on GSE81224. The Chip Analysis Methylation Pipeline (ChAMP) package is a pipeline which not only integrates currently available 450k analysis methods but also offers its own novel functionality. Circular layout (cyclize package) is an efficient way to visualize huge amounts of genomic information.

### Human tissue samples, cell lines

This study was approved by the institutional review board of The First Affiliated Hospital of University of South China and informed consent was obtained from all patients included in this study. Paired fresh OC tissues were collected from 25 patients who underwent OC resection without prior radiotherapy and chemotherapy in The First Affiliated Hospital of University of South China in 2018. These samples were snap-frozen in liquid nitrogen immediately after resection, and then stored at − 80 °C until needed. The SKOV3, OV90, HO8910 and HOSE cell lines were purchased from the BeNa Culture Collection (Shanghai, China). The OV90 and HO8910 cell lines were cultured in DMEM 1640 medium, SKOV3 and HOSE cultured in RPMI 1640 medium (Sigma-Aldrich Corp., St. Louis, MO, USA) containing 10% fetal bovine serum (Invitrogen) and incubated in a thermostat at 5% CO_2_, 37 °C.

### Cell transfection

24 h before transfection, cells in logarithmic growth stage were digested with trypsin and resuspended with complete culture medium. Cell suspension was prepared by blowing and mixing with straw. 1 × 10^6^ cells were seeded in each of the 6-well, and then cultured in incubator at 37 °C and 5% CO_2_ for 18 to 24 h, until cells reached 50–60% of the coverage rate. 3 h before transfection, the original medium was removed and replaced with a fresh basic medium without serum and antibiotics. Using liposome Lipofectamine 2000 (Life Technologies, USA) according to the kit instruction for transfection, and cultured at 37 °C and 5% CO_2_ conditions for 48 h.

### Methylation-specific PCR

DNA extracted from tissue samples and cell lines was subjected to bisulfite modification to convert all unmethylated cytosines into uracils, leaving methylated cytosines unmodified. The bisulfite modification was carried out by using the CpGenome™ DNA modification kit (Chemicon International, Temecula, CA). MSP was performed using AmpliTaq Gold with primers specific for methylated and unmethylated sequences of the genes. MSP primers for each gene were listed in Table 1. The treated DNA was used immediately or stored at − 20 °C until use. The bisulfite modified DNA was subjected to PCR. Positive control methylated DNA samples for each gene examined was used. The conditions of ampify the bisulphite converted DNA by MSP primers was 95 °C 3 min (95 °C 10 s, 60 °C 30 s, 72 °C 20 s) 40 cycle, 72 °C 7 min, 4 °C ∞. Water blank was used as a negative control. PCR products were analyzed on 2.5% agarose gel and visualized under UV illumination.

**Table 1:** Sequences of MSP primers for qRT-PCR

Name	Sequence
MSP primers	
FOXD3 forward	5′ GGTAGCGTTAGCGATATGTTC 3′
FOXD3 reverse	5′ ACGTCGCTATCCTTCTCTTC 3′
Unmethylated primers	
FOXD3 forward	5′ GGTAGTGTTAGTGATATGTTT 3′
FOXD3 reverse	5′ ACATCACTATCCTTCTCTTC 3′

### In vitro epigenetic drug treatment

The human ovarian cancer cell line SKOV3 and OV90 were treated with a culture medium containing a demethylating agent, 5-aza-2′-deoxycytidine (5-Aza-dC; Sigma-Aldrich, USA) dissolved with acetic acid in 1:1 ratio and subsequently added into the medium. And equivalent acetic acid was added into the negative control groups’ medium. For the 5-Aza-dC treatment, 5-Aza-dC (10 μM) was replenished daily for 72 h. The culture medium was altered every day. After harvesting the cells, RNA was extracted as described below for reverse transcription Quantitative real-time PCR (qRT-PCR), and proteins were extracted for the Western blotting analysis.

### Quantitative reverse transcription polymerase chain reaction (qRT-PCR)

The total RNA from cells and tissue was extracted with the mirVana miRNA isolation kit (Ambion, USA) according to the manufacturer’s protocol. The cDNA was synthesized from total RNA using PrimeScript™ RT Reagent Kit with miRNAs specific RT primers (Applied Biosystems, Waltham, MA) (Takara, Dalian, China) in a total reaction volume of 10 μL in TPersonal Thermocycler (Biometra, Göttingen, Germany) by following the manufacturer’s instructions. Then, miRNA cDNA was quantified using SYBR Premix Ex Taq kit (Takara, Dalian, China) in a 20-μL reaction system (Applied Biosystems, Foster city, CA). The relative expression levels were evaluated by using the by 2^(−∆∆Ct)^ method. Primers for each gene were listed in Table 2.

**Table 2:** Sequences of primers for qRT-PCR

Name	Sequence
FOXD3 forward	5′-GACGACGGGCTGGAAGAGAA-3′
FOXD3 reverse	5′-GCCTCCTTGGGCAATGTCA-3′
β-Actin forward	5′-GGACTTCGAGCAAGAGATGG-3′
β-Actin reverse	5′-AGCACTGTGTTGGCGTACAG-3′

### Western blotting

Western blotting was performed in tissue samples or cultured cells as indicated. The cells were lysed in buffer containing 1% NP40, 50 mM Tris, 5 mM EDTA, 1% sodium deoxycholate, 1% SDS, 1% Triton X-100, 10 mg/mL aprotinin, 1 mM PMSF, and 1 mg/mL leupeptin (pH = 7.5), supernatants were collected after spin and protein was measured by Bradford assay (Thermo, Waltham, MA, USA). Forty micrograms total proteins were resolved on SDS-PAGE. Following an electric transfer onto PVDF membranes, the blots with proteins were then blocked by 5% bovine serum albumin and incubated with appropriate primary antibodies at 4 °C overnight. The membranes were incubated by HRP conjugated secondary antibody, and signals were visualized by an enhanced ECL-based imaging system. The caspase 3 detected was active caspase 3. Antibodies used in the study include anti-FOXD3 antibody (ab178512, Abcam, USA), anti-beta Actin antibody (ab8227, Abcam), anti-Annexin A2 antibody (ab41803, Abcam), anti-Cleaved PARP1 antibody (ab4830, Abcam), anti-Casepase-3 antibody (ab13847, Abcam), and anti-GAPDH antibody (ab9484, Abcam). The graphs shown the representative images from three independent experiments. The results were compared with normal human ovarian cell line HOSE.

### MTT assay

Cells were prepared into single cell suspensions with culture medium containing 10% fetal bovine serum. After 48 h of incubation at 37 °C and 5% CO_2_ in the constant temperature incubator, 10 μL MTT solution was added (5 mg/mL PBS, pH = 7.4) into each well. After incubate for 4 h, careful remove the culture supernatant. 100 μL medium was added into each hole and oscillate for 10 min to dissolve the crystal. 450 nm was selected to measure the light absorption values for each hole at the enzyme-linked immunoassay. The results were recorded and repeated three times.

### Wound healing test

For wound healing test, the cells were plated in 6-well plates. The adherent cells were wounded by a 10 μL plastic pipette tip and then the scathing cells were rinsed with PBS and cultured with serum-free DMEM for another 24 h. There were four groups, including control group, p-vector group, p-FOXD3 group, and 5-Aza-dC group, where p-vector and p-FOXD3 represented empty plasmid vector group group and FOXD3 overexpression group respectively in the whole article. The wound closure in different groups was photographed and evaluated with the microscope. Finally, the wound healing area after 48 h was analyzed and statistically analyzed. The results were repeated three times.

### Colony formation assay

SKOV3 and OV90 cells were re-suspended and seeded in 12-well plates at a density of 2000 cells/well, incubated for 2 weeks, and then stained with 0.5% crystal violet for 30 min. Excess dye was rinsed off twice with phosphate-buffered saline (PBS). Images were obtained using the computer software Quantity One^®^ from Bio-Rad Laboratories, Inc.

### Flow cytometric assays for apoptosis and cell cycle

For cell cycle, cells were digested by trypsin/EDTA (Gibco) and washed twice with ice-cold PBS. After fixed with 70% ethanol overnight at 4 °C, the cells were washed twice with PBS and then digested by 50 mg/mL RNase in 500 mL of PBS at 37 °C for 30 min. Next, the cells were stained with 20 mg/mL propidium iodide (PI) for 30 min at 37 °C. Finally, FACS Calibur flow analyzer (BD, USA) was used to detect the cell cycle, and FACS Diva (BD, USA) software was used to analyze the data. The experiment was repeated three times.

For apoptosis, endogenous apoptosis of the cells was used to monitor the changes. Cells were harvested using trypsin/EDTA and washed with PBS, and subsequently binding buffer was added to re-suspend the cells. Following incubation with Annexin-V and PI staining according to the manufacturer’s protocol (Bio-Vision), FACS Calibur flow cytometry was used to detect apoptosis, and FACS Diva software was used to analyze data. The experiment was repeated three times.

### Xenograft experiments

6-week-old female BALB/c normal mice and nude mice were obtained from the Animal Center of the Chinese Academy of Medical Sciences (Beijing, China). For tumor-initiating assays, 6 BALB/c nude mice were used every sample. Tumor-initiating cell frequencies were calculated using extreme limiting dilution analysis. Tumor cells (1 × 10^6^) were injected into 6-week-old BALB/c nude mice. All mice were raised for 4 weeks. The mice of 5-Aza-dC group were subcutaneously injected to the posterior flank with 1 mg/kg day 5-Aza-dC for the last 4 weeks every day [14]. From the 1st week, every 5 days, tumor volume and tumor weight were calculated. All protocols for animal studies were reviewed and approved by the Institutional Animal Care and Use Committee of The First Affiliated Hospital of University of South China.

### Statistical analysis

SPSS 19.0 statistical software (Chicago, IL, USA) was used for statistical analysis. Quantitative data were expressed as mean ± standard deviation. A comparison of groups was analyzed by single factor ANOVA and qualitative data were expressed as the number of cases or percentage (%). A comparison of the groups was made using the χ2 test. Statistical significance was set at *p *< 0.05.

## Results

### Genome data analyses for ovarian cancer patients

**Fig. 1:**
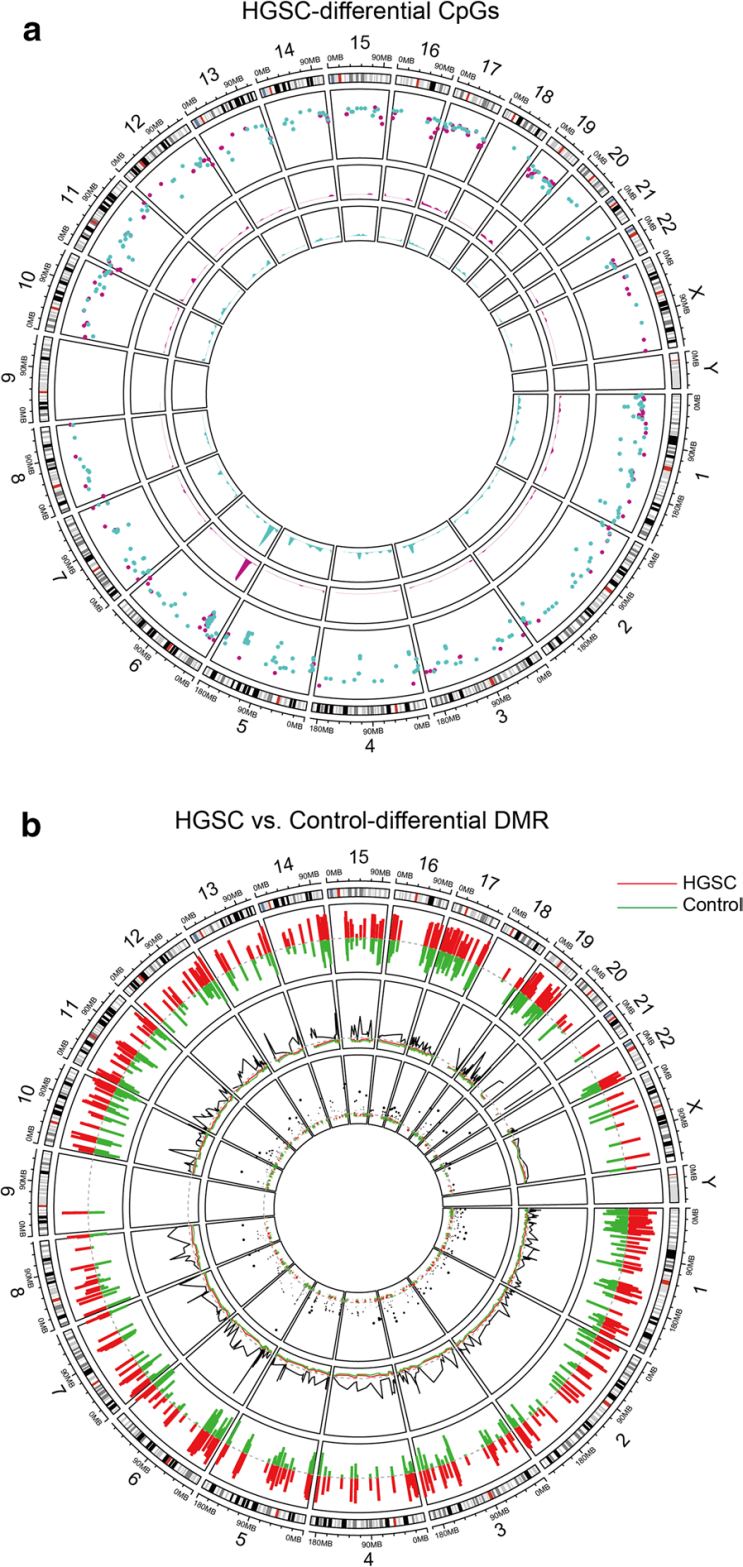
Genome sequencing analysis of ovarian cancer cell. **a** Chromosome analysis of differential CpG sites with ovarian cancer cell from GEO database (ID: GSE81224). **b** DMR analysis between ovarian cancer and normal group. *HGSC* high grade serous ovarian cancer

**Fig. 2:**
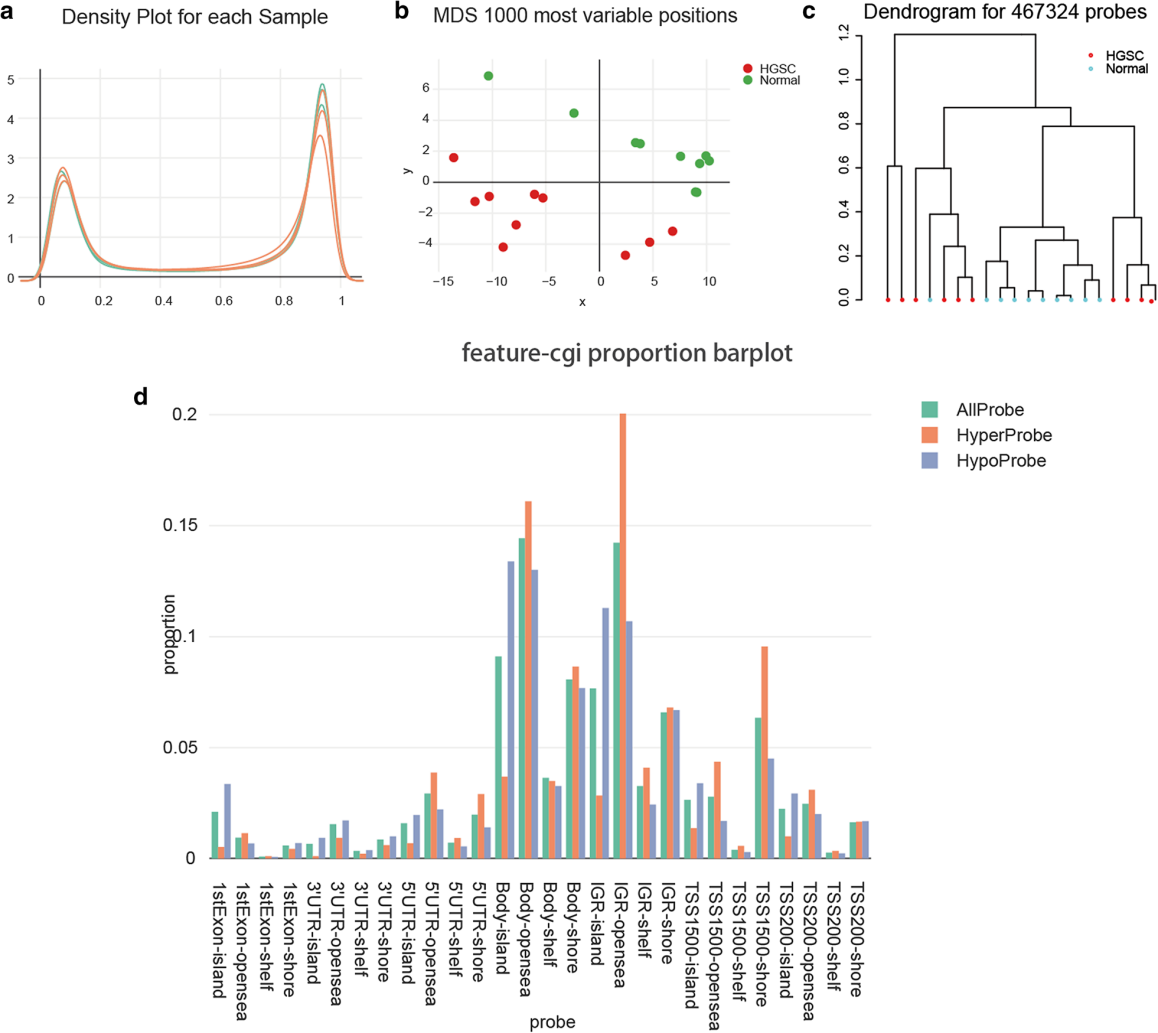
Microarray analyses for ovarian cancer patients. **a** Density of methylated DNA intensity for each sample. The quality of the data for each specimen was visualized by density plot. **b** Multi-dimensional scaling (MDS) plot showed differential clustering of control vs. tumor tissues. **c** Dendrogram produced for 467,324 probes in control and tumor tissues. **d** Genetic and epigenetic annotation information revealed the distribution of top 1000 differentially methylated imprinted CpG sites

We downloaded OC cell chromosome data from GEO database (ID: GSE81224) to analyze differential CpGs and DMR, which showed wide methylation in chromosomes (Fig. 1a, b). We analyzed the genome-wide methylation data of an independent dataset consisting of 10 OC samples. The density plot for each sample was used to confirm performing analysis based on a comparison of beta value density from the rest of the samples (Fig. 2a). We found methylation data was able to discriminate tumors from their surrounding tissues (Fig. 2b). DNA methylation difference of 467,324 probes in control and tumor tissues was detected by hierarchical clustering analysis (Fig. 2b). Genetic and epigenetic annotation information revealed that the distribution of top 1000 differentially methylated imprinted CpG sites (Fig. 2d). Altogether, compared to the overall distribution of all probes, the differentially methylated probes are mainly distributed in the CpG islands.

### *FOXD3* was hypermethylated and low expressed in tumor tissues

**Fig. 3:**
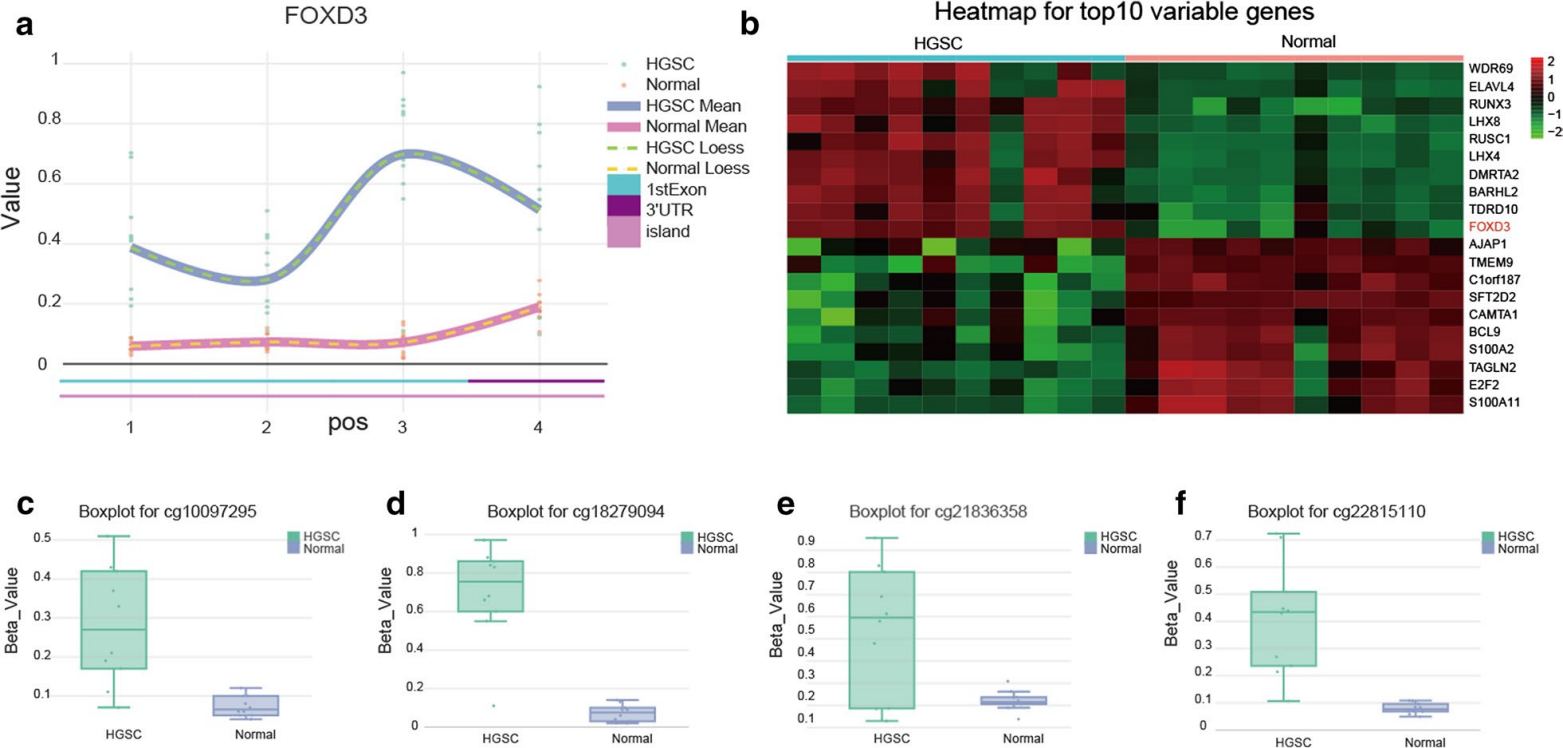
*FOXD3* methylated analysis of tumor tissues and normal tissues. **a** *FOXD3* was differentially methylated in tumor tissues compared to paired normal tissues. **b** The top 20 candidate genes methylation degree were analyzed, *FOXD3* was found to be hypermethylated in tumor tissues compared to paired normal tissues. **c**–**f** Boxplots of methylation data for 4 of the top differentially methylated imprinted sites in the ovarian cancer tumor vs. surrounding comparison (n = 10 tumor/surrounding pairs): **c** cg 10097295, **d** cg 18279094, **e** cg 21836358 and **f** cg 22815110

According to Illumina450 genome-wide methylation data, *FOXD3* methylation in tumor tissues was higher compared to paired normal tissues (Fig. 3a). We also found that *FOXD3* was hypermethylated in tumor tissues compared to normal tissues in the top 20 candidate genes analyzed (Fig. 3b). From the boxplot data of 4 differentially methylated CpG sites, we found an increased methylation in the tumor groups displayed, compared to normal tissues. Boxplot for cg 10097295, cg 18279094, cg 21836358 and cg22815110 were shown (Fig. 3c–f). Altogether, these results suggested that *FOXD3* was hypermethylated in OC tissues.

### Methylation and expression levels of *FOXD3* in various ovarian cells

**Fig. 4:**
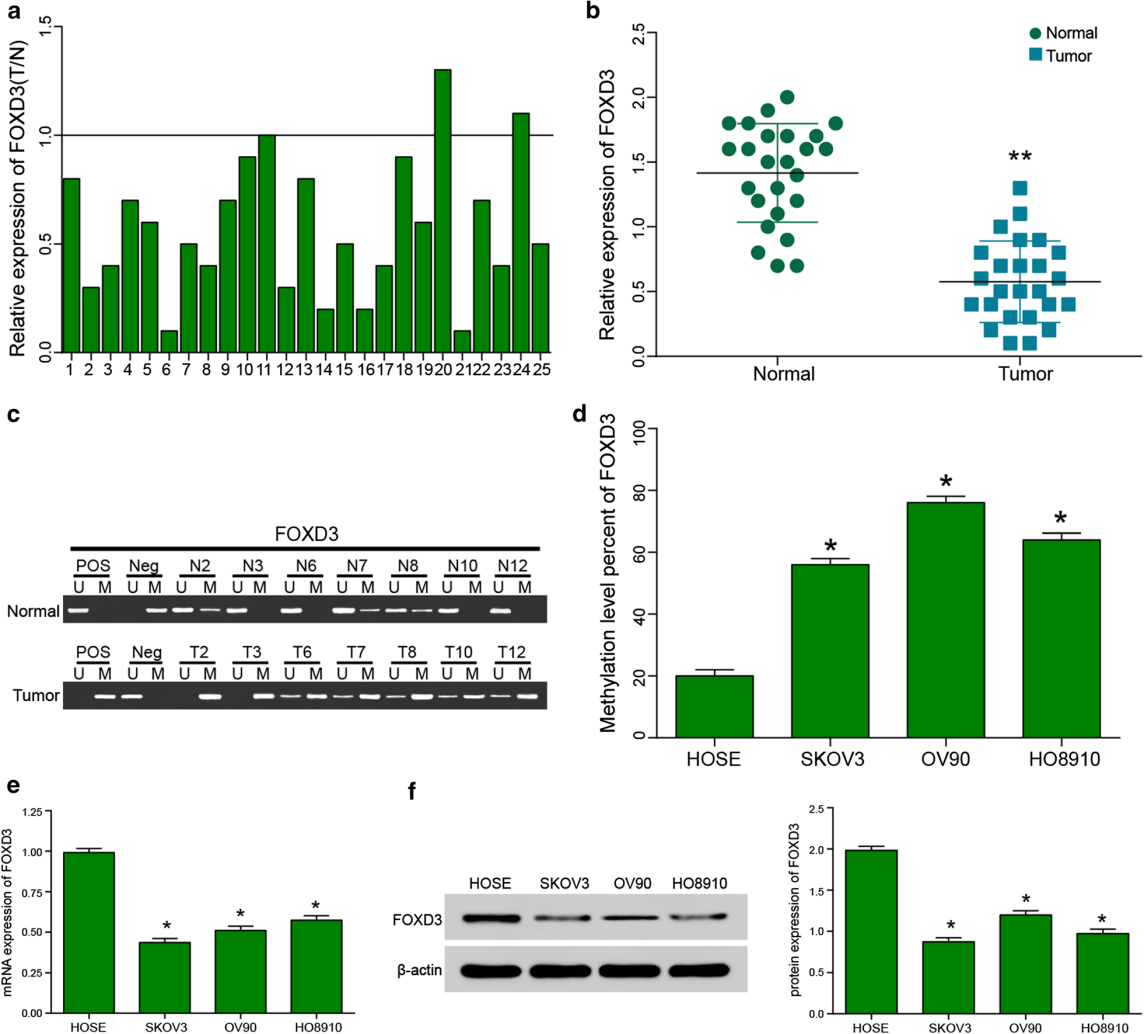
Methylation of *FOXD3* promoter and expression level in various ovarian cell. **a** Real-time PCR analysis showed that *FOXD3* was significantly downregulated in 25 tumor tissues. **b** Compare to normal tissues, *FOXD3* downregulation was statistically significant, *p < 0.05. **c** Methylation-specific PCR showed *FOXD3* degree of methylation in normal tissues and tumor tissues. **d** Compared with that of the HOSE cell line, the *FOXD3* promoter methylation degree of SKOV3, OV90 and HO8910 were significantly increased, *p < 0.05, **p < 0.01. **e** The results of *FOXD3* mRNA expression level by Real-time PCR analysis in SKOV3, OV90 and HO8910 cell lines, *p < 0.05, compared with the HOSE cell line. **f** The results of FOXD3 protein expression level by western blotting in SKOV3, OV90 and HO8910 cell lines, *p < 0.05, compared with the HOSE cell line

The methylation status of *FOXD3* in OC and normal tissues was investigated via DNA methylation analysis. Real-time PCR analysis showed that the *FOXD3* expression level of tumor was significantly higher than that in normal tissues (Fig. 4a, b). These data also showed that *FOXD3* was down regulated in human OC. Compared with normal tissues, the *FOXD3* methylation level in tumor tissues was obviously increased. In three cell lines, the *FOXD3* methylation degree also was significantly increased, compared with that of the HOSE (Fig. 4c, d). Next, the expression of *FOXD3* were detected in SKOV3, OV90 and HO8910 cell lines, and found that the mRNA and protein level of *FOXD3* was down-regulated in three OC cell lines, compared with that in HOSE cell lines (Fig. 4e, f).

### *FOXD3* hypermethylation suppresses ovarian cancer proliferation migration and apoptosis in vitro

**Fig. 5:**
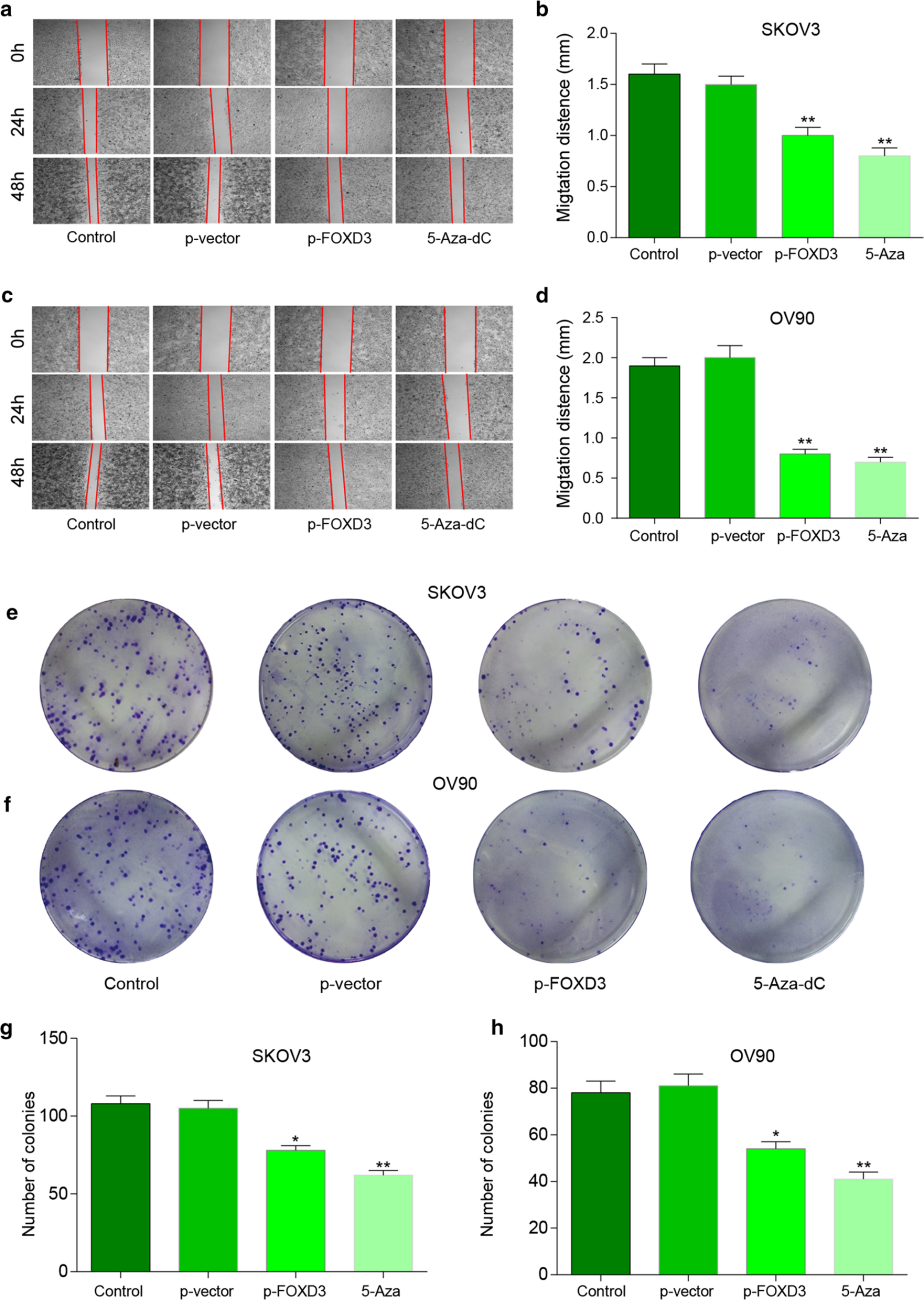
*FOXD3* hypermethylation suppresses ovarian cancer proliferation migration and apoptosis in vitro. **a**–**d** The wound healing test indicated that *FOXD3* over-expression group and 5-Aza-dC group significantly suppressed cell migration ability compared with control group, *p < 0.05. **e**–**h** The colony formation results demonstrated that *FOXD3* over-expression group and 5-Aza-dC group suppressed the level of SKOV3 and OV90 cells colony formation more obviously compared to control group, *p < 0.05

**Fig. 6:**
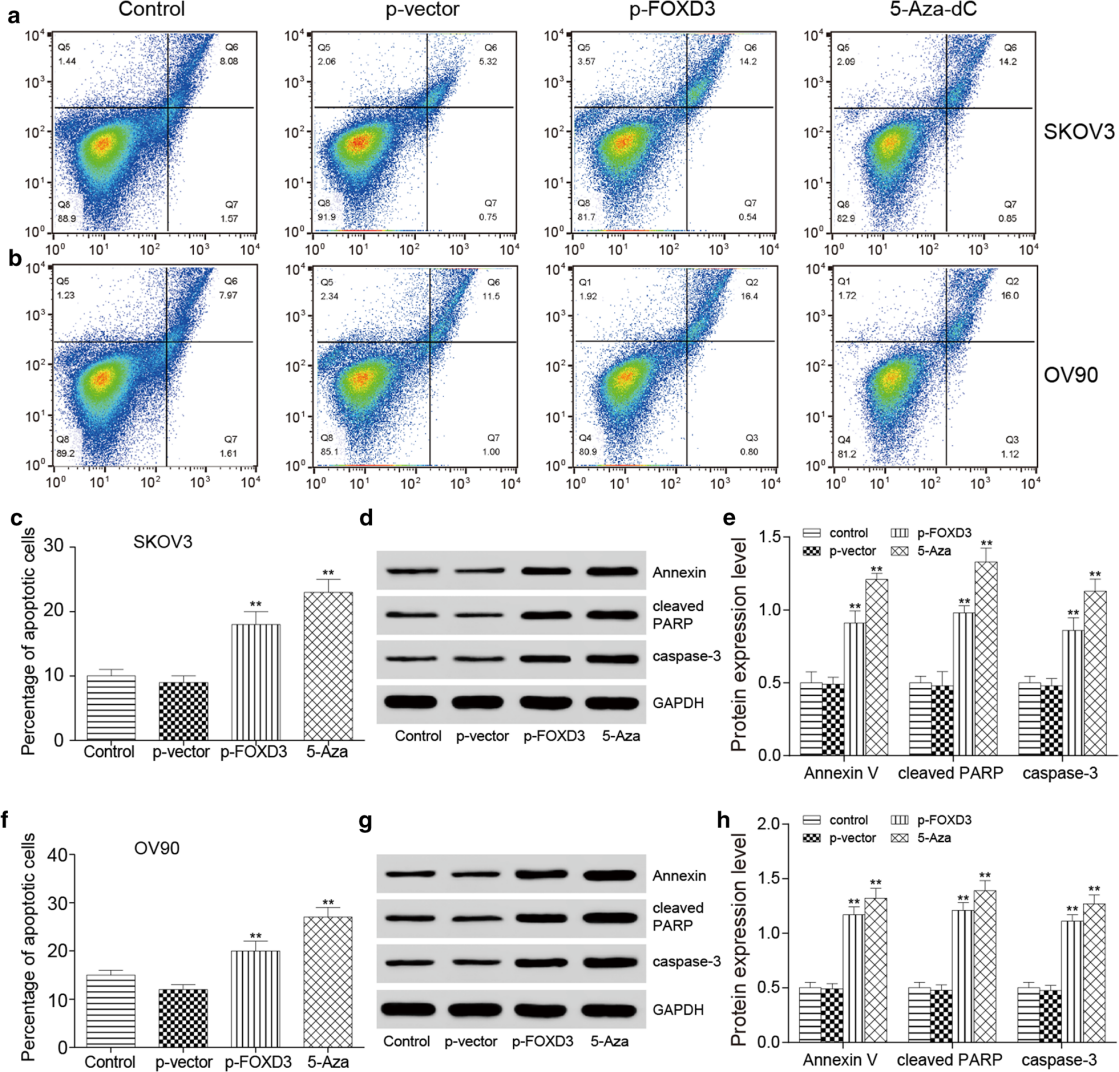
*FOXD3* hypermethylation suppresses ovarian cancer apoptosis in vitro. **a**, **b**, **c**, **f** Flow cytometry showed that the percentage of cell apoptosis elevated in control group, but *FOXD3* over-expression group and 5-Aza- dC group increased the most. The data from flow cytometry confirmed the results, *p < 0.05. **d**, **e**, **g**, **h** Expression levels of apoptotic proteins Annexin V, cleaved PARP and caspase-3 in SKOV3 and OV90 cells. The results showed that the expression level of apoptotic proteins in the over-expression *FOXD3* group and the demethylated group was higher than that in the control group, **p < 0.01

**Fig. 7:**
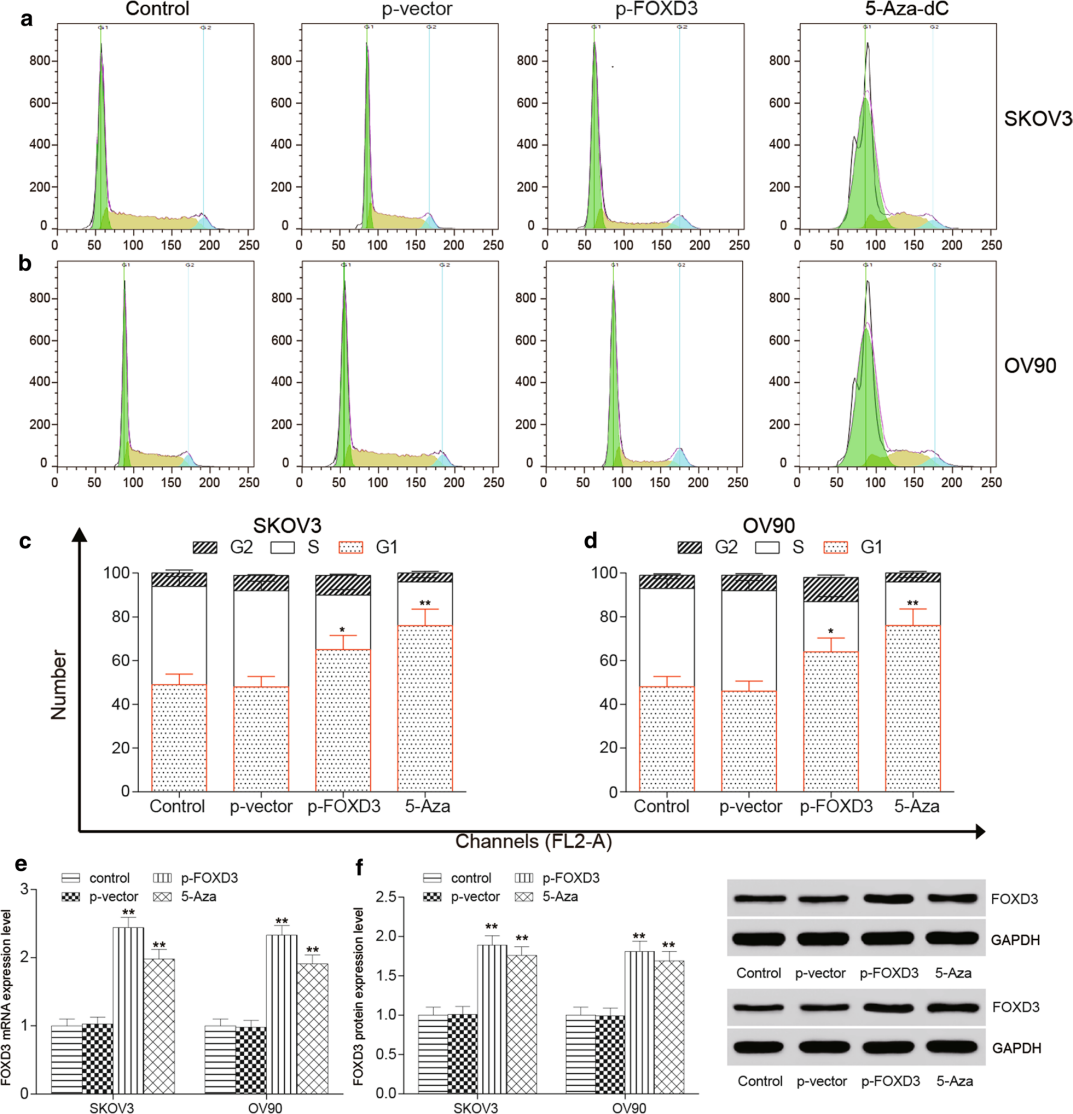
*FOXD3* hypermethylation suppresses ovarian cancer cycle in vitro. **a**–**d** Flow cytometry was performed to detect the cell cycle, the data showed that *FOXD3* overexpression group and demethylation group was significantly longer than that of control group, *p < 0.05. **e**, **f** Expression levels of FOXD3 in SKOV3 and OV90 cells. The results showed that the expression level of SKOV3 in the over-expression *FOXD3* group and the demethylated group was higher than that in the control group

To further investigate whether *FOXD3* affects the progression of OC, we performed anchorage dependent wound healing test, colony formation and flow cytometry assays in SKOV3 and OV90 cell lines. The epigenetic therapy used in 5-Aza-dC group. MTT assay was used to test the effective of 5-Aza-dC in HOSE, SKOV3 and OV90 cell lines. And the minimum effective dose of 5-Aza-dC in SKOV3 and OV90 cell lines was detected and 10 μM concentration of 5-Aza-dC was chosen for minimum effective dose (Additional file 1: Figure S1A, B). The wound healing test indicated that *FOXD3* overexpression and 5-Aza-dC significantly suppressed cell migration ability compared with control group (Fig. 5a–d). Furthermore, the colony formation results demonstrated that *FOXD3* overexpression and 5-Aza-dC suppressed the level of SKOV3 and OV90 cell colony formation more obviously compared to the control group (Fig. 5e, h). Flow cytometry was detected and we found that the percentage of cell apoptosis increased in treatment group (Fig. 6a, b, c, f). Western blot experiment was used to detect the expression level of apoptotic proteins Annexin V, cleaved PARP and caspase-3, and the results showed that the expression level of apoptotic proteins in the over-expression *FOXD3* group and the demethylated group were higher than that in the control group (Fig. 6d, e, g, h), with significant differences. The cell cycle of *FOXD3* overexpression group and demethylation group was significantly longer than that of control group (Fig. 7a–d). These findings suggest that FOXD3 could act as a tumor suppressor to inhibit cell proliferation, migration and promote cell apoptosis in OC cells. In addition, the mRNA and protein expression levels of *FOXD3* in cells of each group were detected by qRT-PCR and western blot experiments. The results showed that the expression levels of *FOXD3* gene in the over-expression group and the demethylation group were significantly higher than those in the control group (Fig. 7e, f).

### *FOXD3* hypermethylation suppresses ovarian cancer growth in vivo

**Fig. 8:**
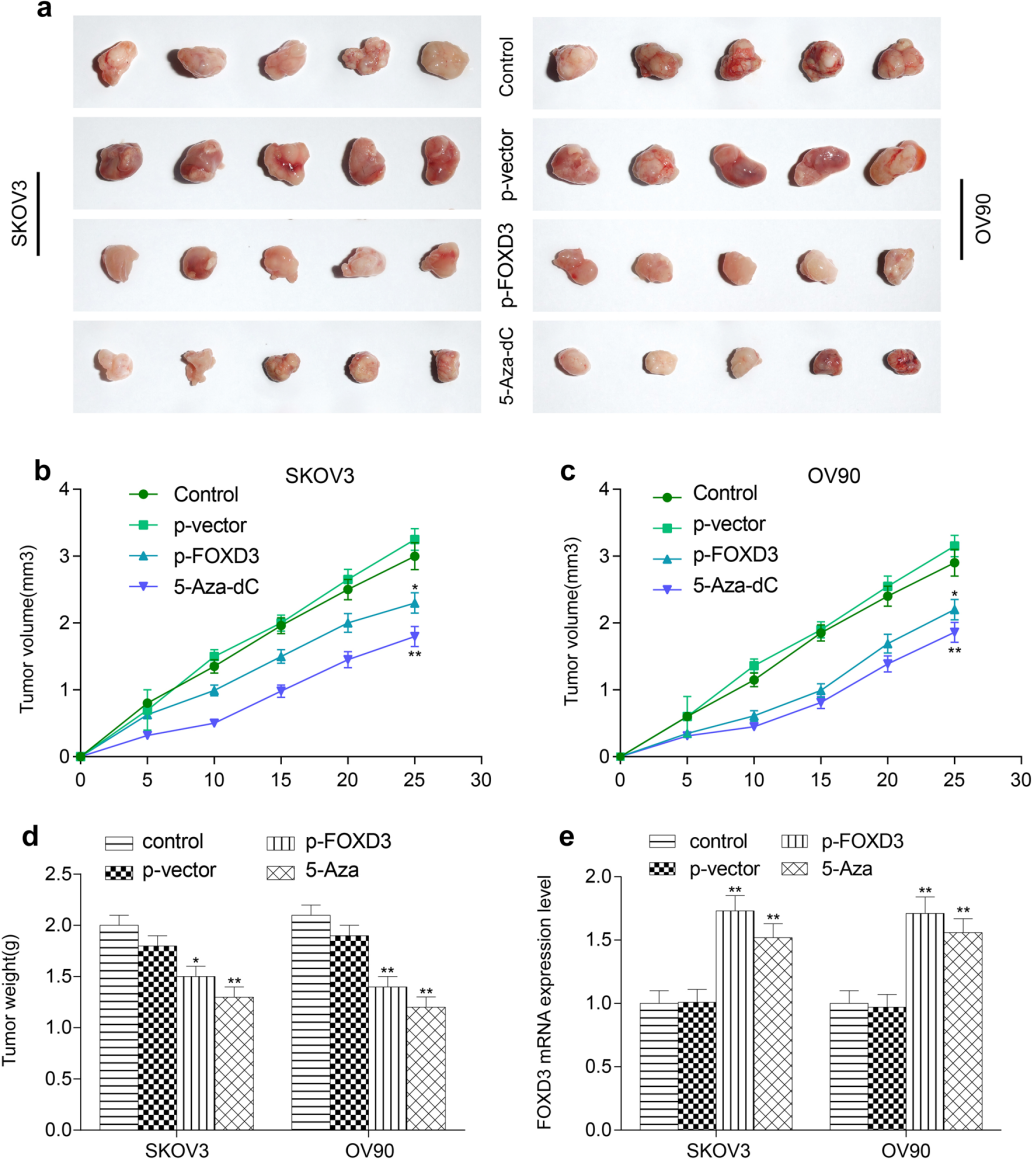
Restored expression of *FOXD3* inhibits the growth of xenograft tumors. **a** Over-expression *FOXD3* group and 5-Aza-dC group showed significantly smaller tumors. **b**–**d** Over-expression *FOXD3* group and 5-Aza-dC group showed obvious inhibition on tumor volume and tumor weight compared with control groups in SKOV3 and OV90 cell lines. **e** Over-expression *FOXD3* group and 5-Aza-dC group showed significantly lower expression level of *FOXD3* in tumors, *p < 0.05, **p < 0.01

To further determine the pathological role of *FOXD3*, we did in vivo xenograft experiments. The epigenetic therapy was used in 5-Aza-dC group and tumors were removed for analysis. As observed by the naked eye, the mice in *FOXD3* overexpression group and 5-Aza-dC group had smaller tumors (Fig. 8a). Furthermore, compared with control group, *FOXD3* overexpression group and 5-Aza-dC group showed obvious inhibition on tumor volume and tumor weight (Fig. 8b–d). qRT-PCR and western blot experiments were used to detect the mRNA expression levels of *FOXD3* in the tumors of each group. The results showed that the expression levels of *FOXD3* gene in the over-expression group and the demethylation group were significantly higher than those in the control group (Fig. 8e). Therefore, restored expression of *FOXD3* also inhibits tumor growth in vivo (Additional file 2).

## Discussion

In this study, *FOXD3* was found hypermethylated in OC tissues compared to normal human ovarian tissues through the analysis of Illumina450 genome-wide methylation data. To further verify the *FOXD3* methylation degree, RT-PCR was performed and suggested that *FOXD3* was hypermethylated and its expression was downregulated in human OC.

DNA methylation is a critical mechanism of gene silence and control of gene expression, which is associated with diverse regulation of cellular process [15]. As a transcriptionally epigenetic modification, abnormal DNA methylation including DNA hypermethylation in CpG islands of promoter regions frequently occur in OC and differ between histological subtypes [16, 17]. It is known that there is a close connection between aberrant DNA methylation and transcriptional inhibition. While hypermethylation in CpG islands of promoters could decrease gene expression, hypomethylation increases gene expression [18]. Thus, the mRNA levels of *FOXD3* detected by RT-PCR could confirm the correlation between *FOXD3* and OC. By figuring out the epigenetic marks that potentially mediated the genetic risk of OC, we could not only better understand the pathogenesis of OC but also enhance the increasing library of risk-related biomarkers for OC [17].

*FOXD3* which primarily identified in embryonic stem cells is a member of the FOX transcription factor family [19]. It is reported that FOXD3 serves as a tumor suppressor in vast types of cancer. Yan et al. found that FOXD3 could suppress tumor growth in non-small cell lung cancer and Li et al. also revealed that FOXD3 could suppress colon cancer [20, 21]. In the present study, we intended to figure out the underling mechanism of *FOXD3* in human OC. Generally, FOXD3 was shown to regulate the downstream microRNA [22]. Combined with western blot, the level of FOXD3 was found to be downregulated in OC cell lines, which indicated that hypermethylation *FOXD3* could associate with a low expression level. Hence the *FOXD3* could serve as a critical factor in human OC.

High levels of promoter DNA methylation that lead to the silence of tumor suppressor genes are resulted from hyperactive DNMTs [23]. DNMTs have been reported to be highly expressed in various types of tumors [24, 25]. Notably, 5-Aza-dC is a DNMT inhibitor which causes a covalent entrapment of DNMT1 to decitabine-substituted DNA and loss of maintenance DNMT activity, subsequently reducing DNA methylation [23]. 5-Aza-dC has also been shown to enjoy the anticancer efficacy on various cancers including ovarian cancer [26, 27]. Furthermore, in order to clarify the pathological role of *FOXD3,* we took 5-Aza-dC as epigenetic therapy on OC cell lines and *FOXD3* overexpression groups used as validation groups. 5-Aza-dC group and *FOXD3* overexpression groups suppressed cell migration and colony formation ability and thus induced apoptotic cell death compared with control group, which suggested that *FOXD3* could inhibit cell proliferation, migration and promote cell apoptosis in OC cell lines. Next, we further verified the pathological role of *FOXD3* in vivo. Similarly, 5-Aza-dC was used as an epigenetic therapy and *FOXD3* overexpression groups used as validation groups, the xenograft experiments revealed an obvious decrease of tumor weight and tumor volume in 5-Aza-dC group and *FOXD3* overexpression groups. These results showed that the restoration of expression *FOXD3* could suppress cancer cells proliferation in vitro as well as in vivo.

## Conclusions

In summary, we firstly found that the hypermethylation of *FOXD3* in OC by bioinformatic analysis. Next, we found that the reversed expression of *FOXD3* in OC could suppress tumor growth. However, in current study there are fewer detected patients in total, so we need to increase the number of samples to confirm our results. In conclusion this study suggested that the *FOXD3* could act as a potential therapeutic target in OC diagnosis and treatment.

## Additional files

**Additional file 1: Figure S1.** Minimum effective dose of 5-Aza-dC was determined. (A–C) Minimum effective dose of 5-Aza-dC was determined by MTT in SKOV3 cell line (A), OV90 cell line (B), and HOSE cell line (C), 10 μM showed difference. *p < 0.05, **p < 0.01, compared with the 0 μM group.

**Additional file 2.** MSP primer sequence corresponds to gene sequence map.

## Abbreviations

ChAMP: The Chip Analysis Methylation Pipeline
DNMTs: DNA methyltransferases
EOCs: epithelial ovarian cancers
DMR: differentially methylated regions
MSP: methylation-specific PCR
OC: ovarian cancer
PBS: phosphate-buffered saline
PI: propidium iodide
qRT-PCR: quantitative real-time PCR
5-Aza-dC: 5-aza-2′-deoxycytidine
