# Supplementary figures and images for: FOXD3 may be a new cellular target biomarker as a hypermethylation gene in human ovarian cancer
Source: Cancer Cell Int. 2019 Feb 28;19:44. doi: 10.1186/s12935-019-0755-8 (PMC6394078; doi:10.1186/s12935-019-0755-8)

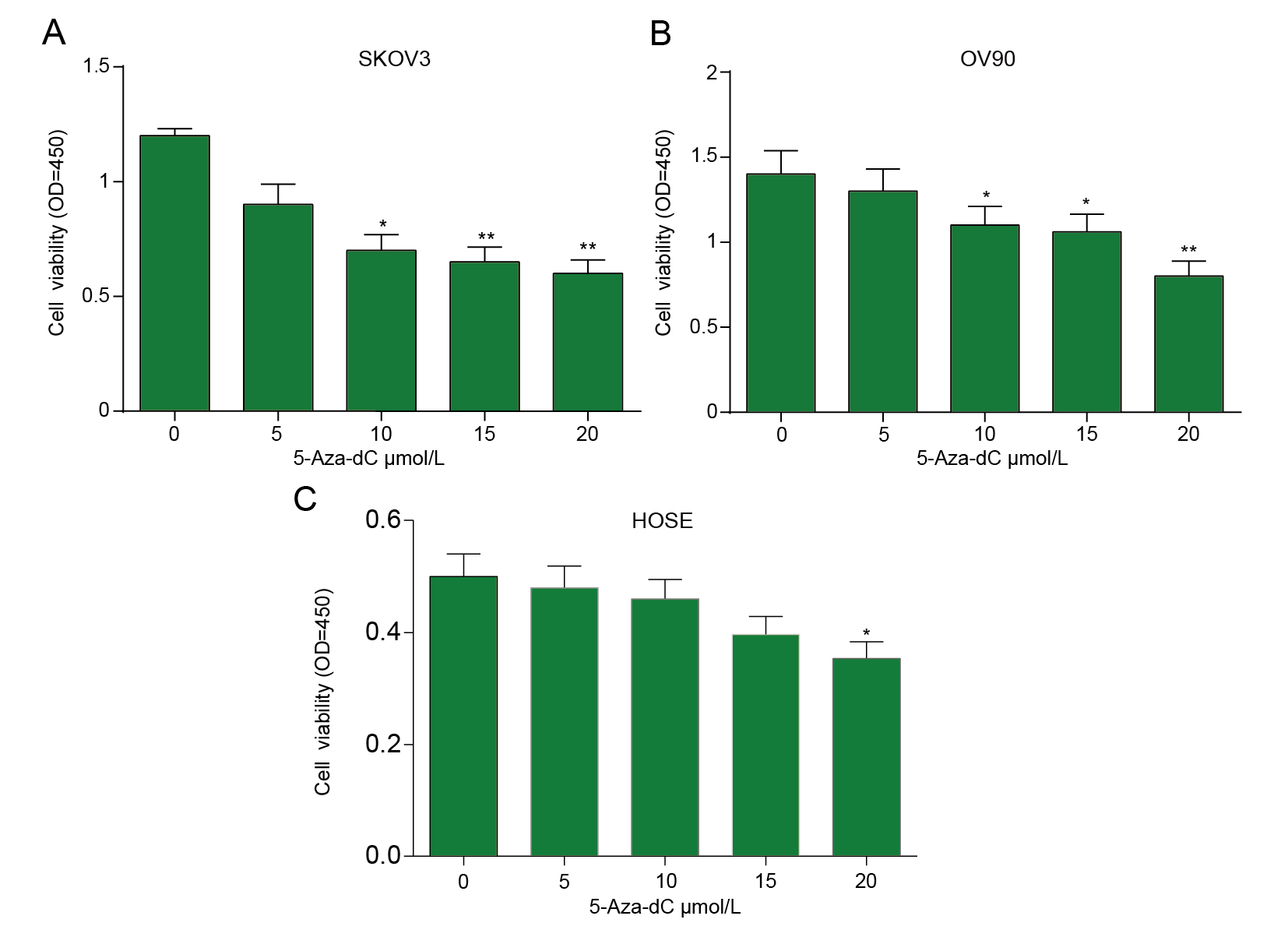

Supplement: Supplementary file 1 — Additional file 1: Figure S1. Minimum effective dose of 5-Aza-dC was determined. (A–C) Minimum effective dose of 5-Aza-dC was determined by MTT in SKOV3 cell line (A), OV90 cell line (B), and HOSE cell line (C), 10 μM showed difference. *p < 0.05, **p < 0.01, compared with the 0 μM group. [file 12935_2019_755_MOESM1_ESM.tif]

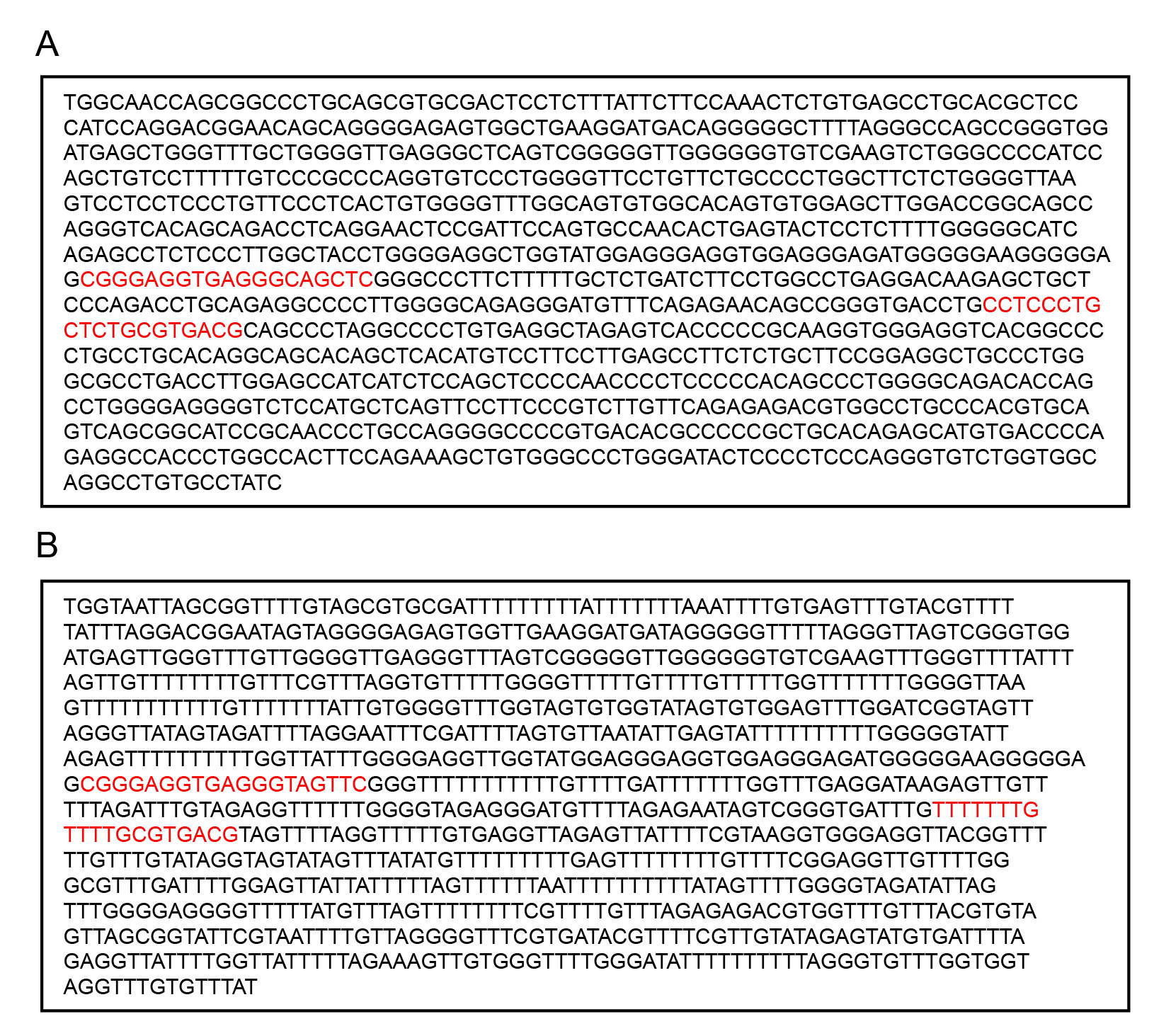

Supplement: Supplementary file 2 — Additional file 2. MSP primer sequence corresponds to gene sequence map. [file 12935_2019_755_MOESM2_ESM.tif]
